# Method for Dairy Cow Target Detection and Tracking Based on Lightweight YOLO v11

**DOI:** 10.3390/ani15162439

**Published:** 2025-08-20

**Authors:** Zhongkun Li, Guodong Cheng, Lu Yang, Shuqing Han, Yali Wang, Xiaofei Dai, Jianyu Fang, Jianzhai Wu

**Affiliations:** Agricultural Information Institute, Chinese Academy of Agricultural Sciences, Beijing 100081, China; 82101232489@caas.cn (Z.L.); chengguodong@caas.cn (G.C.); yanglu01@caas.cn (L.Y.); hanshuqing@caas.cn (S.H.); wangyali01@caas.cn (Y.W.); 82101235610@caas.cn (X.D.); 821012410534@caas.cn (J.F.)

**Keywords:** dairy cow, lightweight model, YOLO v11, target detection, multi-target tracking

## Abstract

With the development of precision livestock farming, in order to achieve the goal of fine management and improve the health and welfare of dairy cows, research on dairy cow motion monitoring has become particularly important. In this study, considering the problems surrounding a large amount of model parameters, the poor accuracy of multi-target tracking, and the nonlinear motion of dairy cows in dairy farming scenes, a lightweight detection model based on improved YOLO v11n was proposed and four tracking algorithms were compared. Firstly, the Ghost module was used to replace the standard convolutions in the YOLO v11n network and a more lightweight attention mechanism called ELA was replaced, which reduced the number of model parameters by 18.59%. Then, a loss function called SDIoU was used to solve the influence of different cow target sizes. With the above improvements, the improved model achieved an increase of 2.0 percentage points and 2.3 percentage points in mAP@75 and mAP@50-95, respectively. Secondly, the performance of four tracking algorithms, including ByteTrack, BoT-SORT, OC-SORT, and BoostTrack, was systematically compared. The results show that 97.02% MOTA and 89.81% HOTA could be achieved when combined with the OC-SORT tracking algorithm. Considering the demand of equipment in lightweight models, the improved object detection model in this paper reduces the number of model parameters while offering better performance. The OC-SORT tracking algorithm enables the tracking and localization of cows through video surveillance alone, creating the necessary conditions for the continuous monitoring of cows.

## Simple Summary

In order to realize the automatic monitoring of dairy cows, this study uses videos of different time periods of dairy farms as a dataset to train a target detection model of dairy cows and compare the performance of different tracking algorithms. In this study, the YOLO v11n model was improved by introducing the Ghost module and the ELA (Efficient Local Attention) module, which improved the performance of the model while reducing the model parameters. At the same time, the SDIoU loss function was used to train the different target sizes in the dairy farm to improve the training accuracy of the model. The model parameters are reduced by 18.59% without pruning operation. The mAP@50, mAP@75, and mAP@50-95 were increased by 0.6%, 2.0%, and 2.3%, respectively. At the same time, this paper compares four different tracking strategies, and the results show that OC-SORT has better performance with a potential HOTA of 89.81%.

## 1. Introduction

As of January 2025, China has approximately 6.159 million dairy cows, with an average of 521 cows per farm, indicating a trend toward large-scale dairy farming. This trend underscores the critical need for digital transformation within the animal husbandry sector. Computer-vision-based animal monitoring has emerged as a key tool for improving efficiency [1,2,3]. By using cow target detection based on a convolutional neural network and the characteristics of a recurrent neural network, the corresponding data can be effectively and continuously extracted [4]. Cow tracking aids in identifying estrus, illness, and daily behavior (feeding, drinking, lying), which are crucial for herd management [5,6,7]. Video-based cow movement tracking enables non-contact monitoring, reduces labor costs, and ensures real-time accuracy [8,9,10]. However, the practical deployment of existing methods faces significant challenges due to the inherent complexity of farm environments and constraints imposed by limited hardware performance.

In some object tracking tasks, they adopted the detect-and-track framework. Among many target detection networks, the YOLO series of models are particularly favored in agricultural applications due to their effective balance of speed and accuracy. Wang et al. [11] used YOLO v8 to obtain information on the behavior of multiple dairy goats in accurate real-time. Paneru et al. [12] chose the YOLO v11 model for chicken detection in a cage-Free flock due to its high portability. When it comes to dairy cows, prior work using the YOLO series of models has shown effectiveness in cow detection through structural improvements and modules. Bai, Q et al. [13] introduced Global Context Block (GC Block) and Res2 backbone to improve the YOLO v3 detection model’s ability to locate cows and detect behaviors. Mon et al. [14] employed the YOLOv8 model to detect a cow in a video, which demonstrated impressive speed, surpassing YOLOv5, Faster R-CNN, and EfficientDet. The above research improves the accuracy of target detection through different versions of the YOLO algorithm, and improves the algorithm by using more advanced modules, so that the model has better detection performance on the dataset. However, some studies did not consider the practical applications of actual scenarios or the impact of model size on hardware. For practical implementation, the key requirements are a small model parameter count coupled with high detection accuracy [15]. Addressing this, our study employs the advanced YOLOv11 model [16], integrating the C3K2 blocks, SPPF, and C2PSA modules to achieve a favorable balance between accuracy and computational efficiency.

Multi-object tracking algorithm performance determines the continuity of target localization. Tu et al. [17] proposed multi-object tracking (MOT) pigs’ behavior based on YOLOv5 fusion Byte to detect individual pig behaviors, and they used the Byte method to track the behaviors of individual pigs, which overcame issues such as false positives, false negatives, and identity switches in the complex environment of pig farms. Li, Q et al. [18] integrated the Dual-YOLOX-Tiny and ByteTrack models to obtain the multi-target pig-video tracking algorithm Dual-YOLOX-Tiny-ByteTrack (DYTB), which addresses the challenges of target detection and tracking in pig farming. Han et al. [19] improved the performance of the DeepSORT-based tracking algorithm for cow tracking to solve scale variations, random motion, and occlusion challenges in cow tracking. These contributions encompass the introduction of a fixed appearance model (AM) capable of accommodating scale deformation, the integration of a five-dimensional ensemble Kalman filter as a motion model (MM) to effectively adapt to unexpected cow movements, and the development of an innovative BM (bench-matching mechanism) designed to handle occlusion. Zheng et al. [20] improved the YOLO v7 algorithm to solve uneven spatial distribution and target scale change in dairy cows, and improved the Kalman filter by directly predicting the width and height information of the tracking frame to improve the ByteTrack algorithm and track cows. These studies demonstrate that combining YOLO variants with tailored tracking algorithms can achieve varying levels of success in livestock scenarios, highlighting the importance of selecting algorithms that are suited to specific deployment contexts. However, much of this research focuses primarily on improving DeepSORT and ByteTrack variants, overlooking the potential application of other advanced MOT algorithms. Given the irregular movement patterns of dairy cows, there may be more suitable tracking algorithms that are yet to be explored for practical deployment.

Aiming at the contradiction between a model’s light weight and accuracy, the adaptability of a tracking algorithm to a cow scene, and the requirements of actual deployed small models, this study proposes a cow tracking method that integrates the target detection of lightweight modules. The GhostConv module is introduced into the backbone network of YOLO v11, and the attention mechanism module is replaced with a lighter module called ELA to decrease the model’s parameters. At the same time, the loss function is replaced to enhance the performance of the model to adapt to the inconsistent target scales of dairy farms. Then, the cow detection box is input into the tracking algorithm, and the ByteTrack, BoT-SORT, OC-SORT, and BoostTrack tracking strategies are systematically compared. These algorithms have different matching rules, and, in practice, the algorithm most suitable for dairy farming scenarios would be selected for application.

## 2. Materials and Methods

### 2.1. Establishment of the Dataset

**Figure 1:**
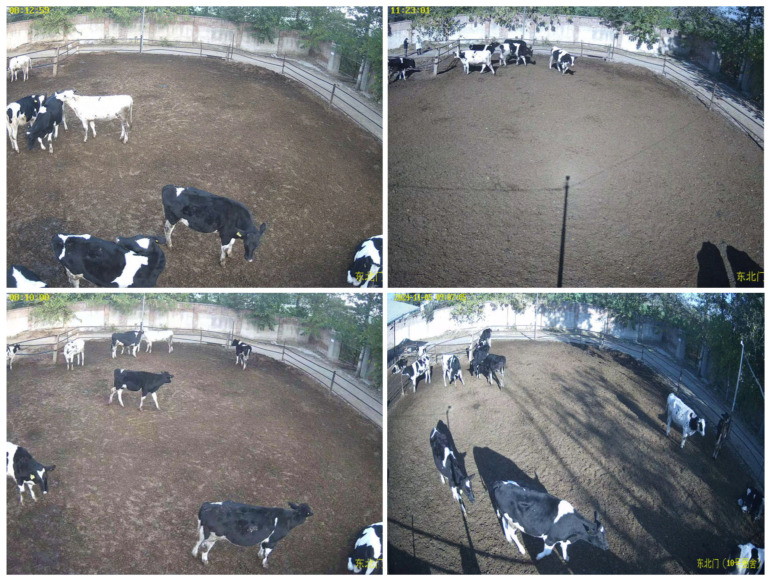
Example images of dairy farm.

The data used in this study were collected from a dairy farm in Beijing Agricultural Machinery Test Station. The camera is installed 4 m high on the south side of the dairy farm, with a top view and backlighting. The videos at 3840 × 2160 resolution and 25 fps were collected on 30 October and 5 November 2024. The data of three time periods were collected in the morning and noon for cow target detection, and two of them were used as a dataset for cow target tracking. Images of the dairy cows were taken, as shown in Figure 1.

**Figure 2:**
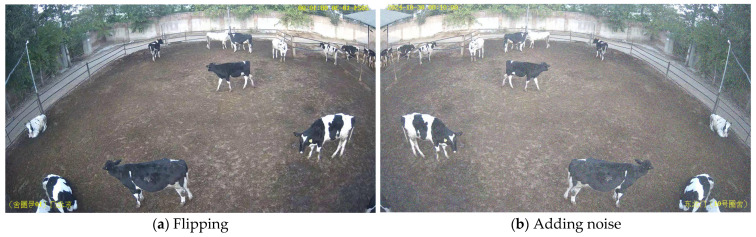

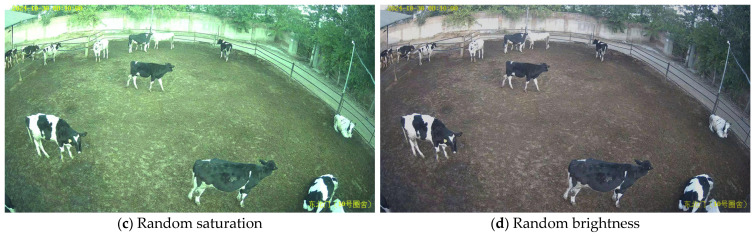
Examples of images obtained after data augmentation.

In this study, videos from two time periods, the morning of 30 October and the noon of 5 November, were collected, which were 3 min 30 s and 6 min long, respectively. The videos were split into frames (JPG format). In total, 950 frames were extracted from these frames, and 526 images from the morning of 5 November were used for detection annotation. A total of 13,260 dairy cows were labeled in these images, with an average of 9 cows per image. Then, data augmentation, including flipping, adding noise, and random adjustments (saturation, brightness), was used to extend the dataset, which improved the generalization ability of the model. A selection of images obtained after data augmentation are shown in Figure 2.

Finally, a total of 7380 images were used as the target detection dataset, labeled by a human using the YOLO format, and divided into training, validation, and test sets in a 7:2:1 ratio. In the meantime, tracking videos were annotated in MOT-20 format using X-AnyLabeling v3.2.0 software, recording individual cow IDs and bounding boxes. The particulars of the dataset are shown in Table 1.

### 2.2. Cow Detection and Tracking Method

#### 2.2.1. YOLO v11 Object Detection Algorithm

The architecture design of the YOLO v11 algorithm combines the dual requirements of lightweight and high performance. Compared to previous versions, the core module improvements include the following: In the Backbone part, the C3k2 (Cross Stage Partial with Kernel Size 2) block is introduced to replace the C2f module of YOLOv8, and the double small convolution kernel strategy is used to replace the single large convolution kernel, and the cross-stage channel separation strategy is used to reduce redundant calculations. This design reduces the number of model parameters, while maintaining the ability to express features; at the same time, the SPPF (Spatial Pyramid Pooling-Fast) module is introduced to enhance the multi-scale target detection ability through multi-scale pooling fusion, and, in particular, the recognition accuracy of small targets is significantly improved. The C2PSA module (Channel and Spatial Part Self-attention Module) is introduced, and the parallel spatial attention mechanism is embedded in the feature pyramid to dynamically allocate the feature weights of different regions.

For the different-sized network models of YOLO v11, in this study, we selected the YOLO v11n model with the smallest number of model parameters, which can have a higher inference speed. We tried to reduce the number of model parameters while maintaining high detection accuracy.

#### 2.2.2. Ghost Convolution Module

**Figure 3:**
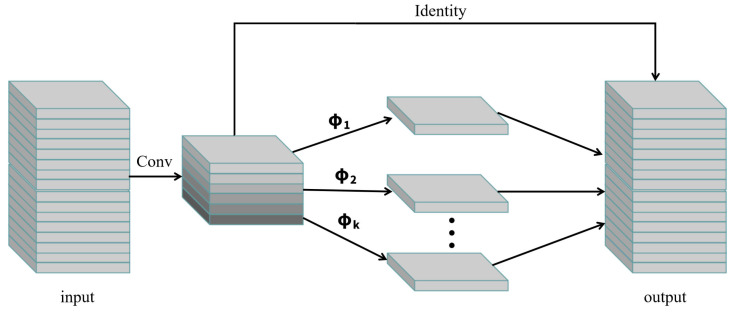
Structure of Ghost convolution module.

Compared to the commonly used convolution kernels, a new Ghost convolution module [21] is proposed in the GhostNet study. Its structure is shown in Figure 3. It innovatively uses the redundancy of the feature map to significantly reduce computational cost by decomposing the convolution process, which provides a new idea for lightweight network design. This module aims to generate more feature maps through cheap operations, generate *m* Intrinsic feature maps through a small amount of conventional convolution (such as 1 × 1 kernel), and then apply *k* groups of cheap linear operations (such as deep convolution) to generate *m* × *k* Ghost feature maps. Among them, the identity mapping preserves the intrinsic features, and the remaining transformations are realized by 3 × 3 small kernel deep convolution. The design breaks through the limitations of conventional convolution schemes: the size of the main convolution kernel can be customized, the channel can be amplified by linear transformation instead of point convolution, and it contains support for diversified linear operations. In the MS COCO detection task, the GhostNet backbone network maintains a mAP value (26.6–26.9%) comparable to MobileNet under the RetinaNet and Faster R-CNN frameworks, which verifies the generalization ability of the module. This work reveals the available value of feature redundancy, and its plug-and-play feature provides an efficient solution for the lightweight of different target detection models.

#### 2.2.3. ELA Module

**Figure 4:**
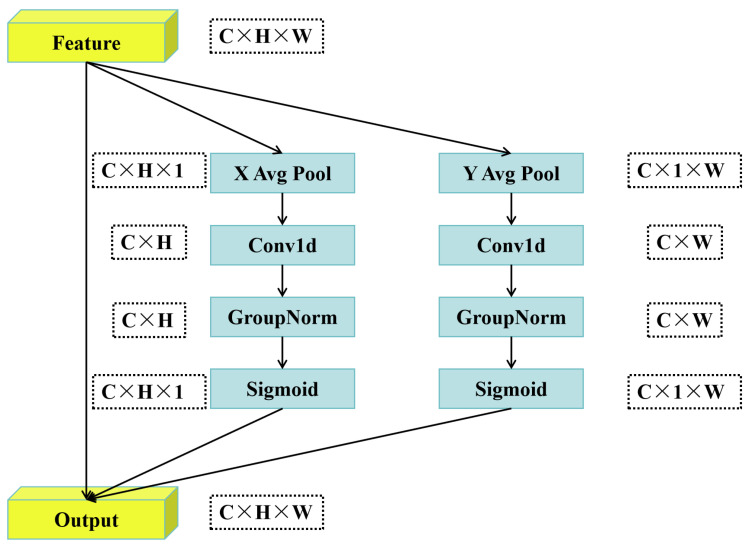
Structure of ELA attention module.

Existing attention methods often face challenges: either it is difficult to effectively utilize key image spatial location information, or else the method is forced to reduce the channel dimension or significantly increase the model complexity when introducing spatial information, which has a potential negative impact on model performance, especially lightweight models. In response to the above problems, we used an efficient local attention mechanism ELA (Efficient Local Attention) [22]; the structure is shown in Figure 4. The core goal of ELA is to accurately locate the ROI (Region of Interest) in the image with a lightweight and concise structural design whilst keeping the channel dimension of the input feature map unchanged. Its design is inspired by an in-depth analysis of the CA (Coordinate Attention) mechanism.

The ELA module adopts a more efficient design strategy: similar to CA, ELA first performs one-dimensional global average pooling (i.e., strip pooling) on the spatial dimensions (height *H* and width *W* directions) of the input feature map to obtain two one-dimensional feature vectors (C × *H* and *C* × *W*). This step effectively captures the long-range spatial dependence and contains accurate location information. A one-dimensional convolution operation is performed independently on the above two one-dimensional feature vectors to replace the two-dimensional convolution in CA. It is naturally suitable for processing such serialized signals, and the calculation is more lightweight and efficient. The output of one-dimensional convolution is sent to the GN (Group Normalization) layer for feature enhancement. Compared to BN, GN is not sensitive to batch size, and shows better generalization performance and stability on small models, which effectively solves the bottleneck of BN in CA.

The key advantages of the ELA module are as follows: it avoids channel dimension reduction, keeps the number of feature map channels unchanged throughout the whole process, and maintains the integrity of channel information; the structure is simple, as only one-dimensional convolution and GN are used; and the number of parameters and computational complexity are significantly lower than those of attention modules, such as CA, that require dimensionality reduction or complex operations. It has strong generalization, making it especially suitable for small and lightweight network architectures, and it also performs well in large networks.

#### 2.2.4. Scale Dynamic IoU Loss Function

In the dairy farm, due to the distance from the camera and the horizontal and vertical posture of the cow, different sizes of detection boxes appear when detecting multiple cow targets. When using the traditional IoU loss function, the stability becomes worse, which has a negative impact on the stability and regression of the model. The SDIoU (scale dynamic IoU loss function) [23] is a new loss function proposed for the problem of labeling noise and scale sensitivity in small-target detection tasks. Traditional detection models generally use fixed-weight IoU variant losses (such as CIoU, DIoU), but the actual labeling data show that there are significant fluctuations in the BBox (bounding box) and Mask labels of the target, and the IoU instability of small-scale targets is more prominent. This kind of labeling noise will interfere with the stability of the model regression, especially the detection accuracy of the weak target.

In order to solve the above problems, SDIoU innovatively introduces a scale-aware dynamic weight mechanism. Its core idea is to adaptively adjust the contribution weight of Sloss (scale loss) and Lloss (position loss) (Lloss) according to the actual size of the target. The specific implementation includes the following: in the BBox branch (SDB Loss), based on the ratio of the target bounding box area Bgt to the maximum target size *B_gt max_* (defined as 81 according to the International Society of Optical Engineering standards), combined with the feature map scaling ratio *ROC*, the dynamic coefficient *β_B_* is calculated. The coefficient constrains the fluctuation range by the threshold parameter *δ,* which limits the value of *β* so as to not exceed. The *δ* value was set to 0.5. Finally, the coefficient generates the scale loss weight *β_LBS_* and the position loss weight *β_LBL_*, and the weighted fusion is the final loss. The loss weight is expressed below [23].

*β_LBS_* = 1 − δ + *β_B_* (1)

*β_LBL_* = 1 + δ − *β_B_* (2)

For the Mask branch (SDM Loss), a similar strategy is also adopted. The β_M_ is calculated by the mask area, and the weights *β_L__MS_* and *β_L__ML_* are assigned to form the mask dynamic loss. The weight is expressed below.

*β_L__MS_* = 1 + *β_M_* (3)

*β_L__ML_* = 1 − *β_M_* (4)

This study shows that the use of the SDIoU loss function can improve the adaptability of small targets and the compatibility of large targets. When the target size is small, the Sloss weight, sensitive to fluctuations, is automatically reduced, and the constraint on the center position error is enhanced. When the target size exceeds the threshold, SDIoU degenerates into a standard CIoU, maintaining its ability to fit large targets. At the same time, the calculation of SDIoU loss function is efficient, avoiding complex exponential operation and only linear calculation.

#### 2.2.5. Structure of Lightweight YOLO v11

**Figure 5:**
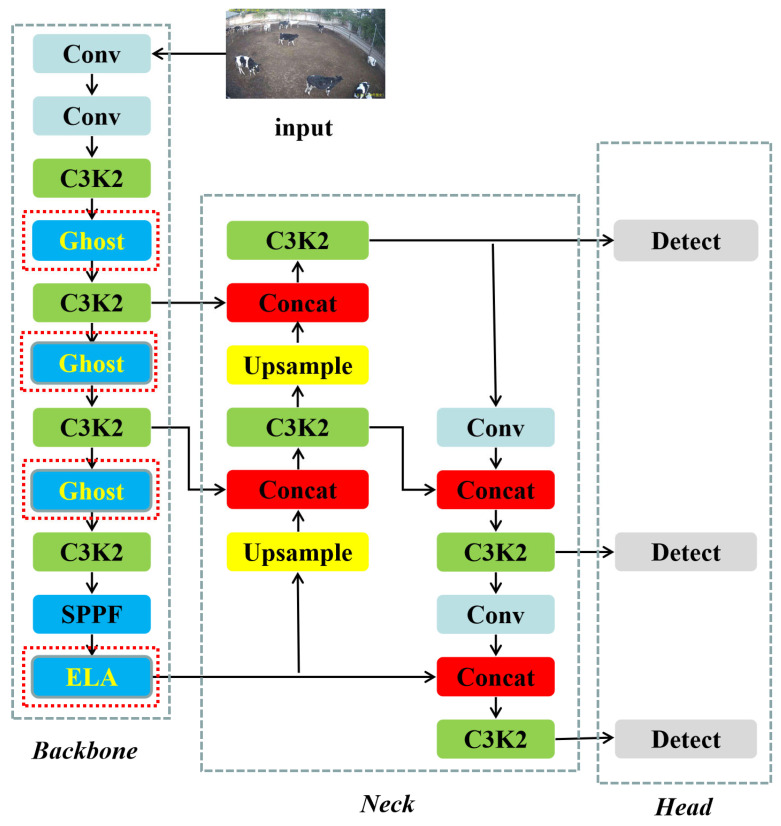
Structure of our lightweight YOLO v11 in this study.

In this study, the above modules are introduced to improve the structure of the YOLO v11 model, as shown in Figure 5. It mainly improves the Backbone part of the model and changes the convolution of the 3rd, 5th, and 7th layers in the Backbone architecture to the Ghost convolution module. By using the redundant information of the feature map, the image features are extracted efficiently, and the original C2PSA attention module is replaced by a lighter ELA module to reduce the number of model parameters. At the same time, in the training process, the SDIoU loss function is used to improve the adaptability and compatibility of different target sizes in the actual breeding scene of dairy cows. With the above improvements, the extracted features are fused to output the detection results.

#### 2.2.6. Cow Tracking Method

ByteTrack [24] can solve a common but easily overlooked problem. Traditional methods usually only associate high-confidence detection boxes (such as IOU greater than 0.5), and directly discard low-resolution boxes. These low-resolution frames actually contain a large amount of real targets, especially occluded or blurred objects. Simply discarding them will result in target loss (missed detection) and trajectory interruption (increased ID switching). Therefore, in the first association, a high-confidence detection box is first used to match the existing target trajectory. The matching basis is usually motion similarity (such as the IoU of the predicted position and the detection frame) or appearance feature (Re-ID), which is similar to the traditional method. In the second correlation, the trajectories that failed to be successfully correlated in the first matching (such as the short disappearance of the target) are matched again with those low-confidence detection boxes. This matching only relies on motion similarity, because the appearance features of the low-score box are usually unreliable.

Bot-SORT [25] has made a number of improvements on the ByteTrack framework. Differently to the traditional method of estimating the aspect ratio of the bounding box, Bot-SORT directly estimates the width and height of the bounding box independently. In order to solve the problem of low overlap between the predicted trajectory and the detection frame due to the movement of the whole picture in the dynamic camera scene (which leads to ID switching or missed detection), Bot-SORT introduces a global motion compensation module. This module uses image registration technology to estimate the background motion (rigid motion) between adjacent frames. Subsequently, the affine transformation is used to correct the trajectory state and its covariance matrix predicted by the Kalman filter so that the prediction box can more accurately correspond to the position of the target in the current frame, which significantly improves the robustness of the motion model in the camera motion or vibration scene.

The OC-SORT [26] algorithm systematically improves the limitations of the traditional SORT tracker in target occlusion and nonlinear motion scenarios. The algorithm deeply analyzes three defects: high sensitivity to state noise, time-domain amplification effect of error during occlusion, and over-reliance on state estimation while ignoring observation data. In order to solve the above problems, OC-SORT innovatively proposes a correction mechanism with observation data as the core. Firstly, using the ORU (observation center re-update) module, the virtual trajectory is constructed by using the first and last observations when the target is re-associated, and the state parameter accumulation error during the occlusion period is corrected reversely. Secondly, the OCM (observation center momentum) module is designed to calculate the consistency of motion direction based on historical observation data and enhance the robustness of data association.

BoostTrack [27] extended the concept of buffered IoU (BIoU) and created a new soft BIoU similarity measure that combines its Mahalanobis distance and shape similarity to discover true positive low-confidence detection. At the same time, a soft detection confidence boost is introduced that considers the original detection confidence score and solves the described problem. For the recently non-updated trajectory, there is usually a relatively low IoU between the matched detection and the matched detection. This problem is solved by introducing different thresholds based on the number of frames since the last update of the given trajectory.

In this study, the above four tracking algorithms were selected for comparison, and the target results detected by YOLO v11 were input for tracking strategy matching. In this study, only motion consistency (IOU) was used for motion trajectory matching to test the matching accuracy of motion consistency of various algorithms.

### 2.3. Experimental Platform and Evaluation Metric

This experiment was conducted using the Windows 10 system. The programming platform used was vscode, and the detail configuration of the server is shown in Table 2.

In the experiment, the YOLO v11 n target detection model was used to train the cow detection dataset, and the input image size was 640 × 640. The image batch size was 32, the learning rate was 0.01, and the model was trained for 200 rounds. Under the premise of ensuring training convergence, the dataset was evaluated. In terms of tracking, the detection threshold was set to 0.6, which requires high confidence of the detection box, and the matching threshold was set to 0.5 to test the matching accuracy of motion consistency (without Reid). The remaining parameters use the default value.

In the target detection part, the accuracy (precision), recall rate (recall), and average accuracy (mAP) indicators were used to evaluate the performance of the model; where mAP took thresholds of 0.5 (mAP@50) and 0.75 (mAP@ 75) and multiple thresholds from 0.5 to 0.95 (step size is 0.05), the average value (mAP @ 50-95) and the model size was evaluated using the parameter quantity (para) and GFLOPs indicators. The evaluation metric is expressed below.

(5) $$P=\frac{TP}{TP+FP}$$

(6) $$R=\frac{TP}{TP+FN}$$

(7) $$AP=\int_{0}^{1}P(R)\cdot dR$$

(8) $$mAP=\frac{1}{N}\sum_{i=1}^{N}{AP}_{I}$$

In the formula, TPs (true positives) represent the number of cows detected correctly, FPs (false positives) represent the number of cows detected incorrectly, FNs (false negatives) represent the number of cows missed, i represents the first category, and N is the total number of categories.

In the multi-target tracking part, the MOTA (multi-target tracking accuracy), MOTP (multi-target tracking accuracyP), MODA (multi-target detection accuracy), ID switching number (IDs), IDF1, and HOTA (high-order tracking accuracy) indicators [28] were used to evaluate the tracker. Among them, MOTA reflects whether there is false detection, missed detection, or identity switching. MOTP is the matching degree of the predicted and actual detection boxes. MODA reflects the performance of the detector. IDF1 is the ratio of the correctly identified detection to the average true number and the calculated detection number. HOTA considers detection accuracy, matching accuracy, and positioning accuracy. The HOTA is expressed below.

(9) $$HOTA=\sqrt{\frac{\sum A(c)}{\left|TP\right|+\left|FN\right|+\left|FP\right|}},A(c)=\frac{\left|TPA(c)\right|}{\left|TPA(c)\right|+\left|FNA(c)\right|+\left|FPA(c)\right|}$$

In the formula, A(c) denotes the correlation accuracy of a single matching trajectory segment, TPA(c) denotes the correct correlation detection point pair, FNA(c) denotes the missed correlation detection point, and FPA(c) denotes the false correlation detection point.

## 3. Results

### 3.1. Cow Target Detection Results and Analysis

**Figure 6:**
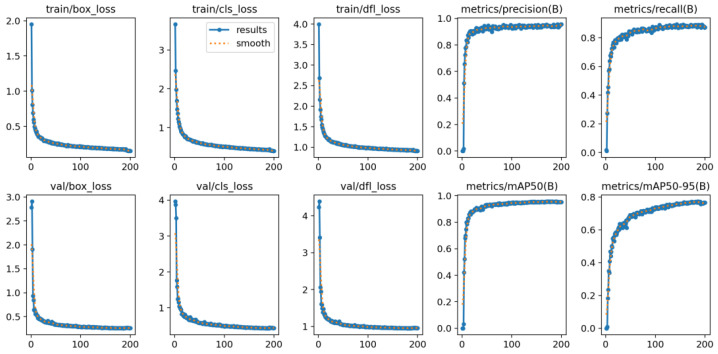
Training process curves.

In this study, the lightweight YOLO v11 model was trained on the cow target detection dataset. The training process is shown in Figure 6.

**Figure 7:**
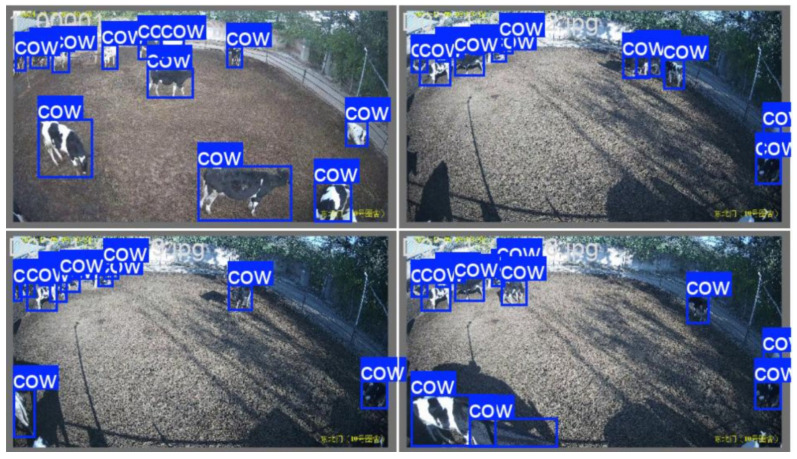
Inferential visualization images for YOLO v11 algorithm.

The main goal of this section is to detect the cow bounding box from the image. Some visual results of the target detection are shown in Figure 7. In order to reduce the confusion of the similarity between the environment and the cow, we did not identify cow targets showing less than three quarters of their whole body in the image.

We selected several classic object detection models, including YOLO v5n, YOLO v10n, and the original YOLO v11n. We used precision, recall, mAP@50, and parameter as evaluation metrics to compare these models with our YOLO model. The results are shown in Table 3.

Compared to the YOLO v5n model, our model performs better in all metrics, with a recall that is 1.6 percentage points higher. Compared to the YOLO v10n and v11n models, the lightweight YOLO v11 model still performs better overall, with a 21.99% and 18.59% reduction in model parameters, respectively. It can be seen that, in the current breeding scenario of dairy cows, the YOLO target detection model has strong robustness and accuracy. In practical applications, the original YOLO model also has good performance, so the lightweight nature of the model has a certain necessity and research value.

In order to verify the effectiveness of the above improvements, the cow target detection dataset was used to perform ablation experiments on the YOLO v11n model and different improved models while ensuring the convergence of model training, and these models were evaluated. The results of each evaluation index of the model are shown in Table 4.

From the results, it can be seen that, after adding three modules, under the condition that the number of parameters is reduced by 18.59% and the calculation amount of GFLOPs is reduced by 0.8 G, the improved model is comparable to the original model. mAP @ 50, mAP @ 75, and mAP @ 50-95 were increased by 0.6%, 2.0%, and 2.3%, respectively. While achieving a lightweight quality, although the accuracy rate P decreased by 0.2%, the overall performance of the model was improved. Among them, the mAP @ 75 and mAP @ 50-95 indicators show a significant improvement, indicating that the improved model has a better detection effect under a higher IOU threshold, more accurate positioning of dairy cow targets, and more stable performance under different strict standards.

In order to verify the effectiveness of the SDIoU loss function used in this study, when other modules are the same, based on the model of YOLO v11n plus the Ghost module and ELA module, the commonly used loss functions (IoU, CIoU, GIoU, DIoU) were used as a comparison with SDIoU. The experimental results are shown in Table 5.

It can be seen from the table that, compared to other loss functions, the overall performance of the model using the SDIoU loss function is better, and the recall rate R, mAP @ 50, mAP @ 75, and mAP @ 50-95 indicators are higher, indicating that the use of the SDIoU loss function can effectively improve the accuracy of target detection and positioning and is more suitable for the cow detection scene in this dataset.

### 3.2. Cow Target Tracking Results and Analysis

In the cow target tracking experiment, the results of the detection box after the target detection are used to compare the different tracking strategies. In the experiment, the tracking algorithm is set to only consider the motion characteristics of the cow, and the experimental results are shown in Table 6.

From the perspective of various indicators, the MOTP index of BoostTrack algorithm can reach 93.09%, but MOTA and IDs perform worse than other algorithms, indicating that the tracking accuracy is poor and the continuity of the cow tracking process is not guaranteed. The MOTA index of the ByteTrack algorithm can reach 96.79%, but the IDF1 is only 84.90%, indicating that the accuracy of the association between the detection box and the trajectory is low, and the continuity of the individual ID of the cow cannot be guaranteed. The BoT-SORT algorithm and the OC-SORT algorithm have better performance in various indicators, but the OC-SORT algorithm performs best. Among them, MOTA, IDF1, and HOTA reach 97.03%, 93.14%, and 89.81%, respectively. Compared to other tracking strategies, the performance is outstanding. At the same time, in the face of long-term video tracking, the number of ID switching is 30 times, which is better than other algorithms.

The reason why the OC-SORT algorithm is better is as follows: Compared to other algorithms, OC-SORT dynamically adjusts the tracking parameters through observation-centered trajectory management, and can better deal with nonlinear motion (turning, sudden acceleration, etc.) of the cow. The virtual trajectory compensation of OC-SORT ensures that the cow has a certain robustness when it is occluded and disappears briefly. At the same time, in the face of the high-frame-rate video (25 fps) in this study, the OC-SORT algorithm is used to reduce the accumulation of cow tracking error and reduce the influence of noise on short-distance displacement.

## 4. Discussion

While prior research has established the value of computer vision for cattle monitoring, achieving a practical balance between computational efficiency suitable for on-farm deployment and robust detection accuracy under variable barn conditions has remained elusive. Our proposed lightweight YOLO v11n model, incorporating GhostConv, ELA attention, and SDIoU loss, successfully navigates this critical tension. Simultaneously, the rigorous comparative evaluation of tracking algorithms provides crucial, operationally relevant insights for real-world implementation.

The developed detector achieves a remarkable 18.59% reduction in model parameters (up to 2.1 million) and a 0.8 GFLOPs decrease compared to baseline. Crucially, this efficiency gain did not compromise core detection accuracy (mAP@50 remained stable at 96.3%), but enhanced performance under stricter localization criteria. The significant 2.0% and 2.3% improvements in mAP@75 and mAP@50-95, respectively, are practically meaningful. They indicate the model’s superior ability to precisely localize cow bodies, even amidst partial occlusions or complex postures frequently encountered in farm settings. This precision is paramount for reliably deriving downstream behavioral metrics essential for farm management, such as accurate posture estimation for resting behavior analysis or robust lameness scoring, directly impacting animal welfare assessment and timely interventions. The synergistic effect of GhostConv (leveraging feature redundancy), ELA (providing focused spatial attention efficiently), and SDIoU (adapting to target scale variation) underpins this success. The minor trade-off in precision (−0.2%) for substantial gains in recall (+2.0%) and higher IoU metrics is strategically advantageous for welfare monitoring, prioritizing the detection of all animals over marginally increased false positives.

From the results of the cow tracking experiments, it is shown that OC-SORT’s superior performance across almost all metrics (MOTA: 97.02%; HOTA: 89.81%; IDF1: 93.14%; IDs: 30) highlights its exceptional suitability for the dairy cow tracking domain. While BoT-SORT also performed well, OC-SORT’s specific innovations directly addressed the core difficulties observed in cow motion. Cows exhibit unpredictable movements, from sudden stops, turns, and accelerations, to changes in gait. Traditional Kalman filter-based trackers (like ByteTrack and, to a lesser extent, BoT-SORT) rely heavily on linear motion assumptions. The Observation-Centric Momentum (OCM) module in OC-SORT, by leveraging historical observation data to assess motion direction consistency, provides inherent robustness against these nonlinearities. It prevents trajectory predictions from diverging rapidly when cows deviate from straight paths.

The combined lightweight detector (YOLO v11n + Ghost + ELA + SDIoU) and OC-SORT tracker offers a highly practical solution for real-world deployment. This is vital for timely interventions (e.g., detecting estrus or early signs of illness). The high tracking accuracy and identity persistence facilitate reliable individual cow monitoring over extended periods, enabling accurate behavior analysis, early disease detection, estrus detection, and welfare assessment. However, this study has limitations that point towards future research avenues. The dataset, although augmented, originated from a single farm. Performance needs to be verified in different environments: different farm structures, different lighting conditions (dawn or dusk), outdoor pastures with complex backgrounds, and different camera angles.

For future studies, in addition to expanding the dataset, cow behavior recognition is also necessary for dairy cow management. Using the tracking results, the algorithm is positioned to further realize the behavior recognition of the cow. Detecting and tracking specific body parts (head, legs, udder) using keypoints would enable more granular behavior recognition (feeding, drinking, lying). Furthermore, video data can incorporate data from other sensors (e.g., RFID for identity confirmation at close range, accelerometers on collars, thermal cameras for fever detection) to create a more robust and comprehensive monitoring system, especially when visual information is compromised. This computer system can significantly contribute to the automation, efficiency, and welfare standards of modern dairy farming. Future work should focus on enhancing generalization, incorporating finer-grained analysis, and rigorously testing the system in diverse operational farm environments to realize its full potential in precision livestock management.

## 5. Conclusions

In this study, we propose a cow target detection and tracking method based on a lightweight YOLO v11n model. By replacing the convolution block with Ghost convolution, using a more lightweight ELA module, and using the SDIoU loss function, we improved the model for the cow target detection scene and actual needs. At the same time, the cow target detection results are continuously tracked using a tracking strategy, and different tracking strategies are compared to achieve multi-target tracking of cows.

The experimental results show that the improved model has better performance while reducing the number of parameters by 18.59% compared to the original model. The mAP@50, mAP@75, and mAP@50-95 increased by 0.6%, 2.0%, and 2.3%, respectively. The SDIOU loss function used is better than several other loss functions and is more suitable for the target detection task of dairy cows. In the target tracking task, the MOTA, MOTP, MODA, IDF1, and HOTA of OC-SORT algorithm are 97.02%, 93.25%, 97.03%, 93.14%, and 89.81%, respectively. Compared to the ByteTrack algorithm, BoT-SORT algorithm, and BoostTrack algorithm, the OC-SORT algorithm has better tracking performance.

We studied the demand for lightweight models in the actual application scenarios of dairy cows and analyzed the more advantageous algorithms in the scenarios of nonlinear motion, frequent occlusion, and high-frame-rate video surveillance of dairy cows by comparing different tracking strategies. This study provides technical support and research ideas for the subsequent positioning and tracking of dairy cows, and has good application prospects.

## Figures and Tables

**Table 1:** Distribution of dataset.

Dataset	Time of Video	Duration of video	Number of Images
A	morning	3:30	1750
B	noon	6:00	3000
C	morning	0	2630
All		9:30	7380

**Table 2:** Experiment environment configuration.

Experimental Environment	Configuration
CPU	Intel^®^ Xeon (R) Gold 6132 CPU @2.6 GHz × 56
GPU	NVIDIA GeForce RTX 2080 Ti (11 G)
Memory	32 G
Computer language	Python 3.10
Deep learning framework	Pytorch 2.3.1
Vision of CUDA	12.1

**Table 3:** Performance comparison of different models.

Model	P/%	R/%	mAP@50/%	Para
YOLO v5n	93.7	89.5	95.8	2181859
YOLO v10n	92.5	88.9	95.5	2694806
YOLO v11n	**94.3**	89.1	95.7	2582347
Lightweight YOLO v11	94.1	**91.1**	**96.3**	**2102315**

**Table 4:** Results of ablation experiment.

Model	P/%	R/%	mAP@50/%	mAP@ 50–95/%	mAP@ 75/%	Para	GFLOPs
YOLO v11n	**94.3**	89.1	95.7	77.2	85.5	2582347	6.3
YOLO v11n+Ghost	93.1	90.5	96.0	77.8	86.5	2348331	5.7
YOLO v11n+ELA	91.4	**91.7**	96.0	78.1	85.8	2336331	6.1
YOLO v11n+Ghost +ELA	93.8	91.1	96.1	78.7	87.0	2102315	5.5
YOLO v11n+Ghost +ELA+SDIoU	94.1	91.1	**96.3**	**79.5**	**87.5**	**2102315**	**5.5**

**Table 5:** Performance comparison of loss function.

Loss Function	P/%	R/%	mAP@50/%	mAP@50–95/%	mAP@75/%
IoU	93.8	**91.1**	96.1	78.7	87.0
CIoU	94.3	90.1	96.1	78.6	86.4
DIoU	**95.0**	88.4	95.8	78.8	87.1
GIoU	93.8	89.6	95.9	78.6	86.9
SDIoU	94.1	**91.1**	**96.3**	**79.5**	**87.5**

**Table 6:** Performance comparison of tracking algorithm.

Tracking Algorithm	MOTA/%	MOTP/%	MODA/%	IDs	IDF1/%	HOTA/%
BoostTrack	90.71	93.09	90.73	42	90.51	85.47
BoT-SORT	96.64	93.20	96.65	31	92.47	89.43
ByteTrack	96.79	92.69	96.82	43	84.90	84.50
OC-SORT	**97.02**	**93.25**	**97.03**	**30**	**93.14**	**89.81**

## Data Availability

The data presented in this study are available on request from the corresponding author due to the privacy of the dairy farm.
